# Impacts of Japanese Larch Invasion on Soil Bacterial Communities of the Giant Panda Habitat in the Qinling Mountains

**DOI:** 10.3390/microorganisms10091807

**Published:** 2022-09-09

**Authors:** Yuqi Zhuang, Yadong Xu, Meiling Yang, Huiru Zhao, Xinping Ye

**Affiliations:** 1 College of Life Sciences, Shaanxi Normal University, Xi’an 710119, China; 2 Research Center for UAV Remote Sensing, Shaanxi Normal University, Xi’an 710119, China; 3 Changqing Field Station for Ecological Research & Education, Shaanxi Normal University, Xi’an 710119, China

**Keywords:** soil bacterial community, soil chemical properties, forest type, high-throughput sequencing, Japanese larch, PICRUSt2

## Abstract

Japanese larch (*Larix kaempferi*), a non-native tree species, has been widely planted in the Qinling Mountains since the last century, but it does not meet the habitat needs of giant pandas (*Ailuropoda melanoleuca*), mainly because of food, further causing habitat degradation and fragmentation. However, how soil microorganisms, considered as predictors of the soil environment, respond to Japanese larch remains poorly explored, especially compared with native forests. Here, we collected 40 soil samples from plantation, bamboo, and natural (excluding bamboo) forests in the Changqing Nature Reserve and Foping Nature Reserve in Qinling to compare soil bacterial community composition and diversity using high-throughput sequencing of bacterial 16S rRNA genes. The soil chemical properties and bacterial communities differed noticeably under forest-type classification patterns. The soil of the Japanese larch planted forests underwent substantial degradation, with higher acidity, lower alpha diversity, and more significant enrichment in the oligotrophic bacteria Acidobacteria and Verrucomicrobia, in contrast to the other two primary forests with elevated soil nutrient levels. The application of PICRUSt2 indicated the down-regulation of amino acid-related metabolism in planted forests. Moreover, pH was the primary factor determining the whole bacterial community structures. To avoid the uncertainty of a single sampling region, we chose different sampling sites that could be considered as geographical factors, possibly due to environmental heterogeneity or dispersal limitations, which also explained the specific community patterns of microorganisms. Overall, this paper may help provide a scientific basis for future revegetation in giant panda habitats, highlighting the urgent need for ecological restoration and sustainable forestry management.

## 1. Introduction

Acting as a critical element of terrestrial ecosystems, soil microbes account for a large fraction of the genetic diversity on Earth [1,2]. In addition, soil microbial communities are pivotal factors in regulating multiple ecological functions and biogeochemical processes [3], including environmental restoration, nutrient acquisition, and carbon and nitrogen cycling [4]. It has been demonstrated that soil microbes tend to be more sensitive to environmental changes compared with other creatures [5], as they are constantly exposed to natural fluctuations linked to plant species, soil characteristics, and geographic distance [6,7], which, to some extent, reflect the reason why soil microbes serve as indicators of forest ecosystem status [8].

There is a growing awareness that plant–microbe interactions make an essential contribution to the ecosystem, implying a solid linkage between the above- and below-ground areas [9]. The most prominent pathways by which trees affect soil microorganisms are through surface litter and root exudates [10]. In a nutshell, plants may cause differences in nutrient inputs into the soil through the variation of both the quantity and quality of litter, thus further influencing the microbial composition and function [11]. For example, microorganisms not only generate a variety of enzymes to hydrolyze and oxidize litter, but also participate in ammonification, nitrification, and nitrogen fixation, which can promote litter decomposition and soil organic matter accumulation [12]. As for the role of root exudates, they are known to mediate plant–microbe interactions, recruiting beneficial microbes or restraining pathogenic ones [13]. In summary, the investigation into the effect of forest type on soil microorganisms should be targeted to specific forest situations for detailed and comprehensive insight.

The Qinling Mountains, a major biodiversity hotspot in China, are renowned for serving as the homeland of giant pandas (*Ailuropoda melanoleuca*) [14]. The larch plantations planted over the last century are severely damaging the quality of the Qinling giant panda habitat, resulting in habitat degradation and fragmentation [15]. In particular, Japanese larch (*Larix kaempferi*), as an exotic species, is one of the typical larches widely cultivated here, drastically restricting the movement of giant pandas and causing soil degradation, especially in contrast to the old-growth forests (e.g., *Bashania fargesii*, *Quercus serrata*, and *Betula albosinensis*); however, there is still a lack of research regarding the variation at the microbiological level. According to relevant studies, giant pandas prefer habitats that preserve their physical fitness and provide high-quality food, and they tend to be found in bamboo forests or larger-sized trees when resting or foraging [16]. Taken together, planted forests are perceived as unsuitable for giant pandas due to sparser trees and lower bamboo coverage, making this a dramatic disruption of habitat connectivity [17]. Moreover, the Chinese government has been committed to the conservation of giant pandas and has proposed the Giant Panda National Park (GPNP) policy for the restoration and connection of fragmented habitats, ultimately achieving a harmonious coexistence between humans and nature [18]. Consequently, we should obtain a thorough overview of the soil bacterial response to Japanese larch invasion, especially compared with local native forests, in order to alleviate the harm caused by invasive exotic tree species.

Here, we sampled from Changqing Nature Reserve and Foping Nature Reserve in the Qinling Mountains to contrast the soil bacterial composition and diversity of planted, bamboo, and natural forests based on bacterial 16S rRNA gene sequencing. Specifically, the objectives of this paper were to: (1) elucidate the alteration in soil characteristics in response to planted and primary forests; (2) investigate the composition and diversity of soil bacteria, contrasting plantation, bamboo, and natural forests; (3) identify soil edaphic factors shaping soil bacterial communities in this unique region; and (4) compare potential functions among the three forest types. The findings of the analysis may enable us to examine the impact of Japanese larch plantations on soil bacterial communities, which can support the subsequent vegetation improvement of giant panda habitats.

## 2. Materials and Methods

### 2.1. Study Area and Sample Collection

Our study was conducted in the habitat of the giant pandas in the Qinling Mountains of Shaanxi Province (106°30′–108°05′ E, 32°40′–34°35′ N), where it is estimated that approximately 350 wild giant pandas are present, from which we chose Changqing and Foping National Nature Reserves, because they are key distribution areas with the highest density of giant pandas, with numbers of 57 and 67, respectively [19]. Changqing National Nature Reserve (107°19′–107°55′ E, 33°17′–33°44′ N) is situated in Yangxian County on the southern slope of the middle portion of the Qinling Mountains, established in 1995. It encompasses approximately 299 km^2^ and ranges from 800 to 3071 m. The average annual temperature is 9.6 °C, and the yearly average rainfall is 814 mm. The vegetation types are complex, mainly cold–temperate coniferous forests in the mid-to-high mountain areas above 2300 m. In comparison, the low and middle mountainous areas below 2300 m are primarily composed of broadleaved forests, interspersed with some coniferous forests and coniferous and broadleaved mixed forests [20]. Foping National Nature Reserve (107°40′–107°55′ E, 33°33′–33°46′ N) covers an area of 292 km^2^, which is located in Foping County, and has an altitude range of 980–2904 m. The average temperature per year is 11.5 °C, and the yearly precipitation is 924 mm. The deciduous broadleaf forests grow below 2400 m, and the vegetation gradually transitions upward to the mixed broadleaf and coniferous forest and the coniferous forest zone at the highest peaks [21]. The soil types are all brown soils.

**Figure 1:**
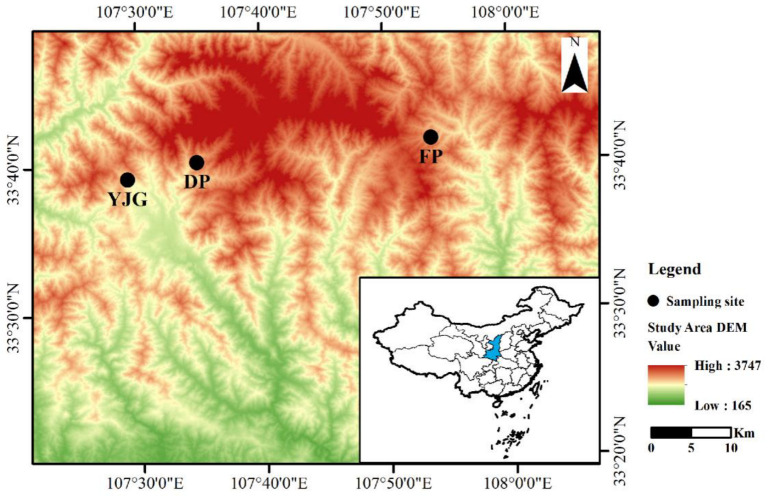
Study sites.

To obtain more representative samples, we sampled from the Yangjiagou (YJG) and Daping (DP) areas of Changqing National Nature Reserve and Foping National Nature Reserve (FP), with average elevations of 1450 m, 1671 m, and 1674 m, respectively (Figure 1). Bamboo forests, such as *Bashania fargesii* and *Fargesia qinlingensis*, are the dominant understory vegetation, which grow at mean elevations of 1600 and 2400 m, respectively [22]. Because of the lower altitude of our sampling sites, roughly around 1600 m, the selected bamboo forest type is *Bashania fargesii*. The main vegetation types of the natural forests in DP and FP consist of deciduous broadleaved forests (e.g., *Quercus serrata*, *Betula albosinensis*, and *Juglans mandshurica*). The bamboo and natural forests were deemed as representatives of native forests. Furthermore, Japanese larch (planted in the 1990s) was chosen as the plantation type. To sum up, among the three areas (YJG, DP, and FP) described above, bamboo forests (B), natural forests (N), and plantation forests (P) were selected as the forest types to be studied (YJG does not include a natural forest here) (Figure S1).

A total of 40 samples of surface soil (0–10 cm) were collected in July 2021; five 10 m × 10 m quadrats were randomly set in each sampling area, with the distance between each quadrat being more than 20 m; thus, the quadrats were considered independent from each other. At each quadrat, five soil subsamples were taken with a spade following an S-shaped curve and were mixed into a combined sample. Then, they were sealed in plastic bags and transported in a refrigerator to the laboratory, where they were immediately processed to remove stones, roots, and litter. Each composite sample was averaged into two identical sections, one of which was stored at 4 °C to be assayed for soil chemical properties, and the other at −80 °C for later DNA extraction and microbial analysis.

### 2.2. Soil Chemical Analyses

Soil pH was measured in a 1:2.5 soil–deionized water (*w/v*) slurry using a digital pH meter (FE28, Mettler-Toledo, Greifensee, Switzerland). The potassium dichromate oxidation method was used to determine the contents of soil organic carbon (SOC, %). Soil total phosphorus (TP, g/kg) and available phosphorus (AP, mg/kg) were separately extracted with NaOH and NaHCO_3_, and then analyzed by the Mo–Sb colorimetric method. The contents of total potassium (TK, %) and available potassium (AK, mg/kg) were determined using flame spectrophotometry (M410, Sherwood, UK) after digestion with HF–HClO4 and ammonium acetate extraction, respectively. The total nitrogen (TN, g/kg) was analyzed by the Kjeldahl method. The soil hydrolyzable nitrogen was measured based on the alkaline hydrolysis diffusion method [23].

### 2.3. DNA Extraction, PCR, and Illumina HiSeq Sequencing

The total DNA was extracted from the samples using the FastDNA Spin Kit for Soil (MP Biomedicals, Kusatsu, Japan) according to the manufacturer’s instructions. The DNA quality was detected utilizing 1% agarose gel electrophoresis and a NanoDrop One spectrophotometer (Thermo Fisher Scientific, Waltham, MA, USA) was used for the concentration and purification of the genomic DNA. After the above operations, the extracted DNA samples were stored at −80 °C before sequencing.

The extracted DNA was amplified by the polymerase chain reaction (PCR) with the primers 338F (5′-ACTCCTACGGGAGGCAGCAG-3′) and 806R (5′-GGACTACHVGGGTWTCTAAT-3′) in the V3-V4 region of the bacterial 16S rRNA genes. A 50 μL PCR mixture was used containing 25 μL of Premix Taq (Takara Biotechnologies, Kusatsu, Japan), 1 μL of both forward primer and reverse primer, 3 μL of template DNA, and 20 μL of ddH_2_O. In a nutshell, after an initial denaturation step at 94 °C for 5 min, the amplification consisted of 30 cycles of denaturation at 94 °C for 30 s, annealing at 52 °C for 30 s, elongation at 72 °C for 30 s, followed by a final elongation step at 72 °C for 10 min, and then the PCR products were maintained at 4 °C. The resulting PCR products were extracted using agarose gel (1%) and further purified with an E.Z.N.A. Gel Extraction Kit (Omega bio-tek, Norcross, GA, USA) following the manufacturer’s protocol. Paired-end sequencing was conducted on an Illumina Miseq platform (Illumina Inc., San Diego, CA, USA).

### 2.4. Processing of Sequencing Data

Paired-end reads of the 16S rRNA gene were demultiplexed and quality-filtered using Trimmomatic (version 0.33, http://www.usadellab.org/cms/index.php?page=trimmomatic; accessed on 27 August 2021) and spliced using FLASH (version 1.2.7, http://ccb.jhu.edu/software/FLASH/; accessed on 27 August 2021) [24,25]. Then, the sequences were assigned to the operational taxonomic units (OTUs) with 97% similarity by UPARSE (version 7.1, http://drive5.com/uparse/; accessed on 29 August 2021) [26]. The taxonomic classification of each representative 16S rRNA gene sequence was annotated and classified using the RDP (Ribosomal Database Project) Classifier algorithm (version 2.2, http://rdp.cme.msu.edu/; accessed on 2 September 2021) based on the Silva database (version 128, https://www.arb-silva.de/; accessed on 2 September 2021) [27].

### 2.5. Statistical Analyses

Alpha diversity, including the Sobs, Chao1, Shannoneven, and Shannon indices, was calculated in QIIME v1.8 (http://qiime.org/scripts/alpha_diversity.html; accessed on 8 September 2021), with rarefaction curves constructed by USEARCH (version 10.0, http://www.drive5.com/usearch/; accessed on 8 September 2021). One-way analysis of variance (ANOVA) was performed with Tukey’s multiple comparisons test at the 95% confidence level. Additionally, two-way ANOVA was applied to examine the effects of the two factors, forest type and site, on the chemical properties and alpha diversity using SPSS Statistics 26.0 (SPSS Inc., Chicago, IL, USA).

The beta diversity of all samples at the OTU level was visualized by principal coordinates analysis (PCoA) to observe the clusters due to differences in the bacterial community structure and was examined by permutational multivariate analysis of variance (PERMANOVA) to evaluate the influence of forest type and site using the “vegan” package in R4.1.0 with the Bray–Curtis distance.

The linear discriminant analysis (LDA) effect size (LEfSe) method (http://huttenhower.sph.harvard.edu/lefse/; accessed on 15 September 2021) was applied to obtain potential microbial biomarkers, which could search for which microbial taxa explain significant differences among the samples in the classification based on forest type or geographical area, calculated in the order of the non-parametric Kruskal–Wallis test, the pairwise Wilcoxon sum-rank test, as well as LDA with a threshold value of 3.5.

The association between microbial community structure and environmental parameters was examined by distance-based redundancy analysis (db-RDA) and the Mantel test, which were implemented using the “vegan” and “ggcor” packages in R software v4.1.0.

### 2.6. Network Analysis

To explore the shared and unique OTUs, a bipartite association network was constructed based on the Spearman’s rank correlations using the “psych” package in R. Cytoscape v3.9.0 was used to implement the bipartite network diagrams using the edge-weighted spring embedded layout algorithm [28].

### 2.7. Functional Prediction Analysis

According to the Kyoto Encyclopedia of Genes and Genomes (KEGG) database, the Phylogenetic Investigation of Communities by Reconstruction of Unobserved States 2 (PICRUSt2) was performed to predict the functional profiles [29]. Subsequent data visualization of the KEGG functional pathways (levels 1 and 2) was carried out using the “ggcor” and “circlize” packages in R.

## 3. Results

### 3.1. Soil Chemical Properties

Among all samples, the pH values of bamboo forests were the highest (Table 1). The bamboo forests had significantly higher values of SOC than the plantations, which only occurred in YJG. From the perspective of different sites with the same forest type, the soils from FP displayed lower concentrations of SOC and HN compared with those of the other areas, while TN was the highest in YJG. The interactive analysis with two-way ANOVA showed that site and vegetation both had highly pronounced influences on pH, and the values of SOC, TN, and HN were all affected by site. Meanwhile, SOC was also influenced by forest type.

### 3.2. Bacterial Community Composition and Diversity

**Figure 2:**
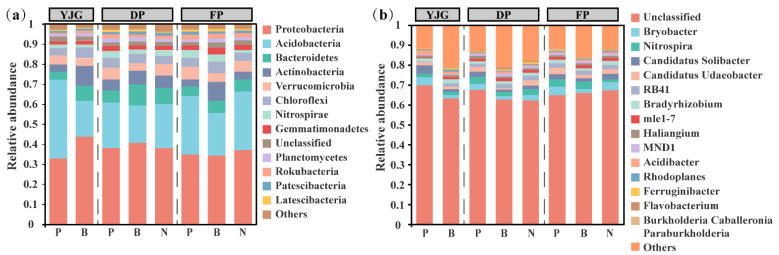
Relative abundance of soil bacterial community structures at the phylum (**a**) and genus (**b**) levels across different soil samples.

The soil bacterial community composition showed a clear distribution, with a total of 42 taxa at the phylum level and 518 taxa at the genus level identified from the 40 samples. Two of the most abundant phyla were Proteobacteria and Acidobacteria, which had relative proportions of 32.84% to 43.86% and 17.79% to 39.27%, respectively (Figure 2a). In all samples, the dominant genera were *Bryobacter* (23.49%), *Nitrospira* (20.56%), *Candidatus Solibacter* (18.29%), *Candidatus Udaeobacter* (15.69%), *RB41* (14.17%), and *Bradyrhizobium* (12.49%) (Figure 2b). It is worth mentioning that Proteobacteria and Bacteroidetes were significantly decreased in the YJG plantation forest, whereas Acidobacteria increased to become dominant.

**Figure 3:**
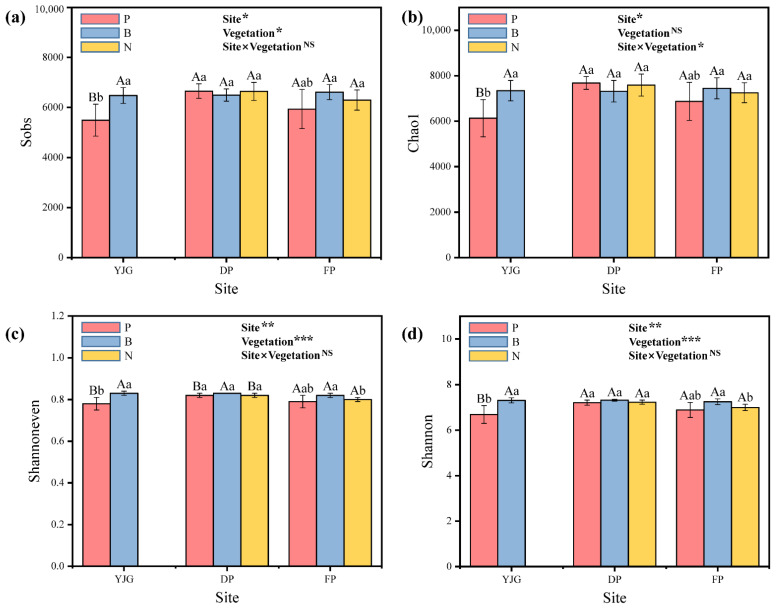
Alpha diversity among different soil samples. (**a**) Sobs. (**b**) Chao1. (**c**) Shannon. (**d**) Shannoneven. Values are shown as the means ± standard error (*n* = 5). Different uppercase letters indicate significant differences between different forest types within the same site, while different lowercase letters represent significant differences between different sites within the same forest type at the 0.05 level. The results of the two-way ANOVA are shown in each subplot. * *p* < 0.05, ** *p* < 0.01, *** *p* < 0.001, and NS = not significant.

The rarefaction curves gradually flattened, but did not reach saturation, suggesting that the sequencing was deep enough to characterize the microbial community with a small number of prokaryotic microbial species that had not yet been collected (Figure S2). The bacterial alpha diversity, assessed by the Sobs, Chao1, Shannoneven, and Shannon indices, varied with forest type and site (Figure 3). Regarding richness, the two-way ANOVA results obtained from the figure illustrate that both the site and forest type significantly affected the Sobs value (Figure 3a), while there was an evident variation in Chao1 by region and region–forest type interaction (Figure 3b). In addition, the Shannon and Shannoneven indices, representing diversity and evenness, respectively, were also strongly influenced by area and forest type, which were accompanied by the more significant impact of the forest type (Figure 3c,d). As for the soil samples from different forest types within the same site, the bacterial richness and diversity indices of the bamboo forest in YJG were considerably higher than those of the plantation. No noticeable differences were observed in the other two locations, except for the Shannoneven index in DP. When comparing all treatments from different geographical areas among the identical vegetation, the plantation soil from DP exhibited a trend with higher values around all alpha-diversity indices in contrast to the other two sites. Distinct from the alpha diversity of plantations, slight, but non-significant, fluctuations can be seen in the bamboo forests. The Shannon and Shannoneven indices of the natural forests tended to be higher in DP than in FP.

**Figure 4:**
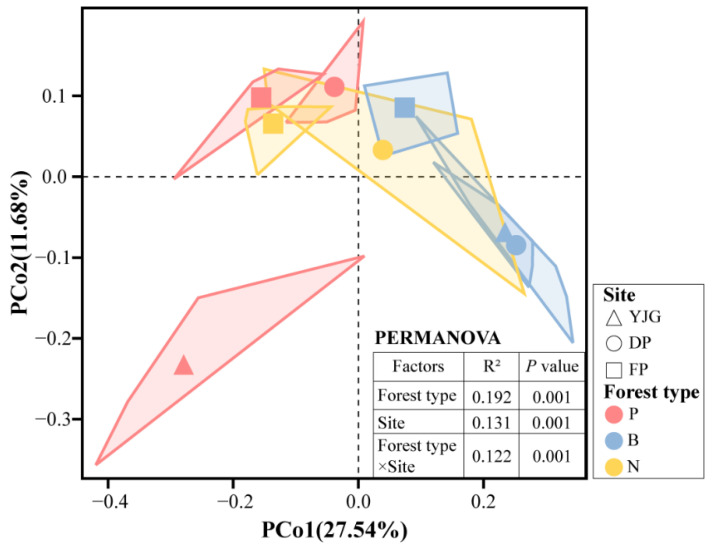
Principal coordinate analysis (PCoA) based on the Bray–Curtis distance of bacterial community compositions.

As demonstrated by the permutational multivariate analysis of variance (PERMANOVA), both forest type and geography caused significant differences in the microbial community compositions, with forest type having a greater impact (Figure 4). To quantify dissimilarity among all soil samples, we used principal coordinate analysis (PCoA) with Bray–Curtis to visualize the bacterial community structure. In general, almost all treatments exhibited a certain degree of similarity, apart from plantation forest in YJG. Analyzed by forest type, plantation and bamboo forests were isolated from each other, and natural forests were partially crossed with both of them. The samples of bamboo forests in YJG and DP were clustered closely. To some extent, the unique bacterial community composition structure of the YJG plantation forest could account for the notable variations in alpha diversity and beta diversity.

### 3.3. Identifying Indicator Taxa among Different Forest Types and Sites

**Figure 5:**
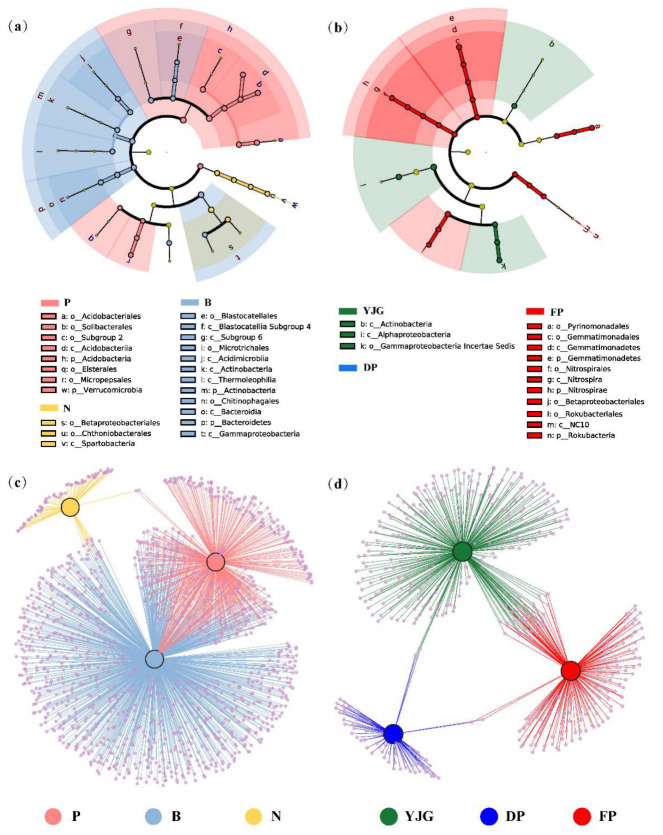
Different characteristics of bacterial communities between different forest types and sites. (**a**,**b**) Cladogram reporting results from the LEFSe analysis for forest types (**a**) and sites (**b**) with a threshold value of 3.5. Circles from inside to outside indicate phylogenetic taxa from phylum to genus, with the taxonomic levels of phylum and order listed in abbreviated form. The diameter of each circle is proportional to the relative abundance. (**c**,**d**) Bipartite association networks revealing the shared and unique OTUs among the three forest stands (**c**) or regions (**d**). The larger nodes represent different treatment groups, and the OTUs, colored in purple, are shown as smaller nodes. The lines (edges) connected to them display significant associations (*p* < 0.05), colored according to the group they belong to.

Linear discriminant analysis (LDA) coupled with effect size measurements (LEfSe) was performed to compare bacterial communities in order to identify the specialized bacterial groups within distinct forest types and sites (Figure 5a,b). In terms of vegetation and region, 35 and 25 bacterial clades, which were considered as potential biomarkers, exhibited significant differences in all soil samples with an LDA threshold of 3.5, respectively (Figure S3). Obvious differences in the soil microbial structure were found, especially between bamboo forests and plantation forests (Figure 5a). The phyla Actinobacteria and Bacteroidetes were enriched in bamboo forests, which included the classes *Acidimicrobiia*, *Actinobacteria*, *Thermoleophilia*, and *Bacteroidia*. Moreover, there was a high abundance of three other classes, *Gammaproteobacteria*, *Blastocatellia Subgroup 4*, and *Subgroup 6*, belonging to bamboo forests. At the phylum level, the plantation forests were enriched with Acidobacteria (class *Acidobacteriia*) and Verrucomicrobia. Only the class *Spartobacteria* with its branch order *Chthoniobacterales* and another order *Betaproteobacteriales* were found to be present in large quantities in natural forests. In addition, the cladogram also identified the potential biomarkers among the YJG and FP soil samples, with no distinct taxonomic differences in DP (Figure 5b). For example, we found that FP soil showed significant enrichment of the orders *Pyrinomonadales*, *Gemmatimonadales* (within the phylum Gemmatimonadetes), *Nitrospirales* (within the phylum Nitrospirae), *Betaproteobacteriales*, and *Rokubacteriales* (within the phylum Rokubacteria). In contrast, the classes *Actinobacteria* and *Alphaproteobacteria* had greater abundance in YJG.

The shared and unique OTUs among the three forest types or regions were further analyzed via bipartite association networks (Figure 5c,d), reflecting the distinct differential clustering. The results of OTU clustering were also roughly similar to those of the LEFSe analysis. On the one hand, the number of unique OTUs of bamboo forests was the largest, followed by plantation and natural forests. On the other hand, the variations in OTUs in geographical aspects could be considered less significant for OTUs than under the forest-type treatment, with more OTUs exclusive to YJG and FP.

### 3.4. Key Factors Shaping Soil Bacterial Communities

**Figure 6:**
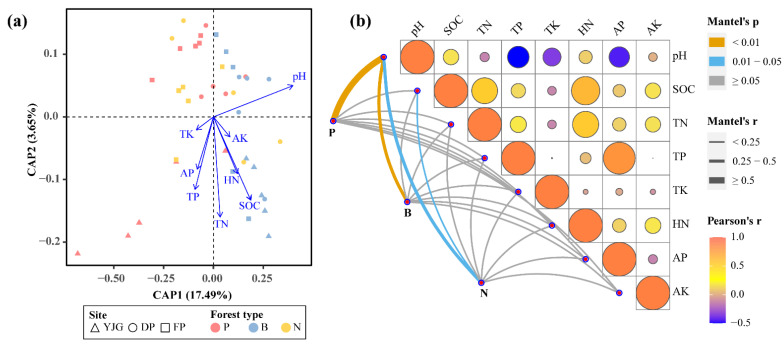
Environmental factor analysis. (**a**) Distance-based redundancy analysis (db-RDA) for the relationship between the soil properties and soil microbial communities across the three forest types from different sites. (**b**) Correlation between the soil bacterial community and soil properties revealed by the Mantel tests.

Distance-based redundancy analysis (db-RDA) was employed to elucidate the relationships between soil properties and soil bacterial communities (Figure 6a and Table S1). The results clarified that the soil pH, SOC, TN, TP, and HN jointly shaped bacterial community structures (Table S1). Furthermore, bamboo forest samples were all distributed in the right half shaft with db-RDA axis-1, which showed that pH, AK, SOC, HN, and TN were positively correlated with bamboo forests (Figure 6a).

In addition, we performed the Mantel tests on the correlations of community structures with environmental factors under the forest type category (Figure 6b and Table S2). The Mantel tests showed that the soil bacterial communities of plantation forests were strongly and positively related to pH. Similarly, the microorganisms of bamboo and natural forests were positively correlated with pH in a relatively weaker association (Figure 6b). Additionally, SOC was also closely associated with the bacteria from the natural forests. Overall, the Mantel tests indicated that pH remained the primary factor determining the whole bacterial community structure, consistent with the db-RDA results; however, it was TP that was the other integral contributor to the entire treatment (Table S2).

### 3.5. Potential Functional Capabilities of Bacterial Communities

The heatmap (Figure S4) reflected the overall functional profiles of level 2 between treatments, yet the clustering of natural forest and plantation forest could be seen. In fact, the results of the one-way ANOVA showed significant differences between the three forest types in terms of amino acid metabolism, biosynthesis of other secondary metabolites, energy metabolism, glycan biosynthesis and metabolism, lipid metabolism, cell motility, and transport and catabolism (Table S3). In particular, there were eight predominant KEGG functional categories at level 2 (relative abundance > 5.0%), accounting for 72.96–73.67% of the total relative abundance, among which amino acid-related metabolisms were strongly down-regulated in planted forests, in contrast to bamboo and natural forests.

## 4. Discussion

### 4.1. pH as the Primary Soil Physical Factor Driving Bacterial Community

Our analysis of soil chemical properties revealed that pH was greatly influenced by forest type, with the highest pH occurring in bamboo forests and the lowest occurring in plantation forests (Table 1). Furthermore, the results of both db-RDA and the Mantel tests indicated that pH was one of the most critical factors regulating soil microbial changes (Figure 6; Tables S1 and S2), which is consistent with previous studies [6,30].

Changes in soil pH due to forest type may have a huge impact on the microbial community [31,32]. Previous studies have found that Pinaceae can promote soil acidification and that long-term monoculture plantations cause similar damage [33,34]. Tripathi et al. [35] stated that soil pH is the best predictor of bacterial community composition and diversity, with the highest diversity occurring at near-neutral pH levels. It is apparent that our results are in accordance with this observation, with the bamboo forests having the closest to neutral pH of around 6.1 and the highest diversity of the three, as opposed to the plantation forests. To account for this phenomenon, it is likely that the soil pH may be directly or indirectly associated as an integrating variable with other soil properties that jointly drive shifts in soil microbes, or that the pH inside bacterial cells is commonly near neutral and the similarity of an external environmental pH value to the intracellular may indicate less energy consumption sustain the internal pH [35,36].

### 4.2. Changes in Bacterial Community Composition and Diversity

As for the alpha diversity, the Shannon and Shannoneven indices, representing diversity and evenness, respectively, characterized different forest types. The values of these two indices decreased in the order of bamboo, natural, and plantation forests (Figure 3). As far as we know, microbial diversity is generally greater in old-growth forests than in plantation forests [37,38]. The establishment of plantations has been subject to plenty of anthropological disturbances, with the monoculture planting of a conifer tree species, Japanese larch, whereas natural forests encompass a more comprehensive range of plant species composed of deciduous broadleaf forests [37]. It has been discovered that litter from broadleaf forests decomposes at a faster rate than that from coniferous forests, accompanied by another theory that the decomposition rate of litter is significantly positively correlated with soil bacterial diversity [39,40]. Moreover, the higher richness of tree species allows for more nutritious litter, which in turn benefits bacteria with diverse living conditions [41]. Yang et al. [42] concluded that the higher intrinsic growth rate of artificial forests interferes more with soil microorganisms, in which case soil bacteria tend to display antagonistic interactions, decreasing microbial diversity. With respect to the bamboo forests, it seems to hold that the increased bacterial diversity can be attributed to the easily decomposable bamboo leaves that act as labile carbon accessible to microorganisms [43].

In terms of bacterial community structures, it can be concluded that the bacterial species composition varied widely between forest types, such as larger proportions of Acidobacteria and Verrucomicrobia occurring in plantation forests, in contrast to bamboo forests, where Actinobacteria and Bacteroidetes were more enriched (Figure 2 and Figure 5; Figure S3). It is worth noting that, among the bacteria described above, Acidobacteria and Verrucomicrobia are known to be oligotrophic bacteria, confirming the assumption that plantation forests are suffering from a relative scarcity of nutrients [44]. Acidobacteria prefer an acidic environment, which is in line with the lowest pH occurring in plantation forests, and are capable of degrading complex biological macromolecules for conversion to active organic carbon and nitrogen sources [12]. Given the fact that the phylum Verrucomicrobia is facultatively or obligately anaerobic, prolonged monoculture of pure larch forests may have led to soil degradation with less porosity and consequently poor permeability, which accounts for the more dominant position of Verrucomicrobia in planted forests [45,46]. With regard to bamboo forests, Actinobacteria, behaving as lignocellulose decomposers, have the capacity to protect the roots from pathogenic microorganisms by producing various antibiotics, consequently boosting tree growth directly or indirectly [47]. Another representative phylum of bamboo forests, Bacteroidetes, is affiliated with copiotrophic bacteria, and participates in nutrient cycling and exhibits excellent denitrification capability [44].

Besides the vegetation factor, the present study revealed that spatial factors also affected soil chemical properties and microbial community to some extent. It is well recognized that soil microbes are susceptible to climatic changes, including temperature and precipitation, while soil chemical properties are also affected by these shifts [48,49]. The yearly average temperature and precipitation in Foping Nature Reserve are higher than those in Changqing Nature Reserve, of which YJG and DP were representative sampling sites. There were much lower contents of SOC and HN in FP, which could be explained by the enhanced soil degradation capacity of nutrients at elevated temperatures [50], and rising rainfall may also have promoted microbial activity and plant nutrient uptake [51]. As for community composition, the phyla Gemmatimonadetes, Nitrospirae, and Rokubacteria, considered to be well-adapted to oligotrophic conditions, were more concentrated in FP, in line with the soil nutrient status of FP, reflecting the variance in the bacterial composition between the two reserves [52,53]. In addition to environmental heterogeneity, dispersal limitation is another crucial factor contributing to the distribution pattern of soil microbial communities, which can be attributed to the integrated effect of niche and neutral theories [54,55].

### 4.3. Comparison of Functional Features between Planted and Native Forests

To gain insight into the functional characteristics of the bacterial microbiota, we conducted PICRUSt2 within KEGG pathways at levels 1 and 2. Our results revealed that the bacterial community as a whole exhibited roughly similar functional characteristics in the three forests (Figure S4), with the highest enrichment of metabolic capacities consistent with previous studies [56], which may be attributed to the similar core functions of different bacteria [57]. Additionally, it is worth noting that the metabolism involved in amino acids was markedly down-regulated in the plantation (Table S3), which reflects the higher turnover efficiency of carbon and nitrogen cycles in primary forests [58]. Owing to long-term monoculture disturbances, it has been challenging to elucidate whether the resistance or resilience mechanisms of soil bacteria contribute to the differences in other functional genes observed in planted forests [59]. Subsequent work is recommended to use metagenomic technology for more accurate functional prediction in conjunction with soil fungi in order to comprehensively assess the soil microbial communities of the giant panda habitat in the Qinling Mountains.

## 5. Conclusions

In summary, soil chemical properties and bacterial community composition were revealed to be notably different when comparing the planted and native forests in the Qinling giant panda habitat. The plantation soils were more acidic, with lower bacterial diversity and higher abundance of the oligotrophic bacteria Acidobacteria and Verrucomicrobia. Meanwhile, the results of PICRUSt2 illustrated a notable down-regulation of amino acid metabolism in the plantation forest. In light of the giant panda conservation ideology, it is imperative to transform planted tree species or build ecological corridors through the rational cultivation of trees and shrubs together with bamboo forests, with the objective of slowing down the fragmentation of giant panda habitats. Overall, this paper may help provide a scientific basis for the future revegetation of giant panda habitats, highlighting the urgent need for ecological restoration and sustainable forestry management.

## Figures and Tables

**Table 1:** Soil chemical properties of the soil samples.

Treatments		pH	SOC (%)	TN (g/kg)	TP (g/kg)	TK (%)	HN (mg/kg)	AP (mg/kg)	AK (mg/kg)
YJG	P	4.87 ± 0.21 Bb	5.62 ± 1.15 Ba	4.1 ± 0.28 Aa	0.43 ± 0.09 Aa	1.14 ± 0.23 Aab	252.42 ± 74.73 Aab	10.12 ± 1.94 Aa	195.8 ± 25.25 Aa
	B	6.11 ± 0.24 Aa	12.4 ± 1.69 Aa	4.54 ± 0.56 Aa	0.3 ± 0.05 Ba	0.99 ± 0.16 Aa	410.93 ± 153.2 Aa	7.12 ± 1.57 Ba	193.2 ± 22.73 Aa
DP	P	5.61 ± 0.1 Ca	6.62 ± 1.85 Aa	3.77 ± 0.63 Aab	0.29 ± 0.12 Aab	1.35 ± 0.2 Aa	362.9 ± 112.93 Aa	7.84 ± 2.71 Aab	195 ± 17.89 Aa
	B	6.1 ± 0.07 Aa	5.63 ± 3.54 Ab	3.42 ± 0.53 Ab	0.28 ± 0.08 Aa	1.05 ± 0.28 Aa	214.52 ± 99.17 Ab	6.02 ± 1.55 Aa	205.8 ± 22.2 Aa
	N	5.83 ± 0.19 Ba	4.18 ± 1.83 Aa	3.5 ± 0.43 Aa	0.26 ± 0.09 Aa	1.07 ± 0.39 Aa	262.5 ± 47.92 Aa	7.32 ± 1.87 Aa	177.4 ± 9.53 Aa
FP	P	5.77 ± 0.15 Ba	2.84 ± 0.52 Ab	3.2 ± 0.35 Ab	0.2 ± 0.05 Bb	0.96 ± 0.06 Ab	112.47 ± 60 Bb	5.4 ± 1.28 Bb	186.8 ± 16.15 Aa
	B	6.09 ± 0.18 Aa	3.67 ± 1.24 Ab	3.43 ± 0.28 Ab	0.33 ± 0.04 Aa	0.94 ± 0.16 Aa	207.33 ± 28.48 Ab	8.02 ± 1.25 Aa	183 ± 17.09 Aa
	N	5.66 ± 0.18 Ba	3.83 ± 1.53 Aa	3.53 ± 0.43 Aa	0.24 ± 0.03 Ba	1.16 ± 0.21 Aa	184.18 ± 53.6 ABb	6.46 ± 0.99 ABa	188 ± 18.8 Aa
Two-way ANOVA									
Forest type		***	**	NS	NS	NS	NS	NS	NS
Site		***	***	***	*	NS	***	*	NS
Forest type × Site		***	***	NS	**	NS	**	**	NS

P (Plantation forests); B (Bamboo forests); N (Natural forests). Values are shown as the means ± standard error (*n* = 5). Different uppercase letters in the same column indicate significant differences among different forest types within the same site, while different lowercase letters represent significant differences among different sites within the same forest type at the 0.05 level. SOC, soil organic carbon; TN, total nitrogen; TP, total phosphorus; TK, total potassium; HN, hydrolyzable nitrogen; AP, available phosphorus; AK, available potassium. * *p* < 0.05, ** *p* < 0.01, *** *p* < 0.001, and NS = not significant.

## Data Availability

Not applicable.

## Supplementary Materials

The following supporting information can be downloaded at: https://www.mdpi.com/article/10.3390/microorganisms10091807/s1, Figure S1: photographs of the sampling sites of the habitat of the giant pandas in the Qinling Mountains, Shaanxi Province; Figure S2: Rarefaction curves of OTUs at 97% similarity for the 40 soil samples. P (Plantation forests); B (Bamboo forests); N (Natural forests); Figure S3: Histogram of the linear discriminant analysis (LDA) effect size (LEfSe) showing taxa with log10 (LDA scores) ≥ 3.5 for forest types (a) and sites (b). The taxonomic levels from phylum to genus are labeled; Figure S4: Heatmap representing the top 20 KEGG pathway level-2 categories under forest type classification via PICRUSt2, with the inner loop indicating pathway level 1, to which they belong; Table S1: Correlation between bacterial community and soil environmental parameters by db-RDA analysis. *p* values in bold mean that the divergence is significant at the 0.05 level; Table S2: Correlation between bacterial community and soil properties as shown by the Mantel test. Bold means that the divergence is significant at the 0.05 level; Table S3: Predicted top 20 level 2 metabolic pathways of the bacterial community based on KEGG. Bold means that the divergence is significant at the 0.05 level. Different uppercase letters indicate significant differences between different forest types.
