# Supplementary material for: Impacts of Japanese Larch Invasion on Soil Bacterial Communities of the Giant Panda Habitat in the Qinling Mountains
Source: Microorganisms. 2022 Sep 9;10(9):1807. doi: 10.3390/microorganisms10091807 (PMC9500889; doi:10.3390/microorganisms10091807)
Supplement: Supplementary file 1 [file microorganisms-10-01807-s001.zip › Tables S1-3.pdf]

Table S1 Correlation between bacterial community and soil environmental parameters by db-RDA analysis. *P* values in bold means the divergence is significant at 0.05 levels.

Soil chemical properties	CAP1	CAP2	R^2	*p* value
pH	0.945	0.327	0.707	**0.001**
SOC	0.759	-0.651	0.435	**0.001**
TN	0.244	-0.970	0.275	**0.006**
TP	-0.407	-0.913	0.237	**0.011**
TK	-0.771	-0.637	0.029	0.597
HN	0.770	-0.638	0.169	**0.028**
AP	-0.567	-0.824	0.098	0.144
AK	0.995	-0.103	0.024	0.663

Table S2 Correlation between bacterial community and soil properties as shown by the Mantel test. The bold means the divergence is significant at 0.05 levels.

Soil chemical properties	r	*p* value
All environmental characteristics	0.203	**0.022**
pH	0.443	**0.001**
SOC	0.087	0.142
TN	0.021	0.328
TP	0.247	**0.007**
TK	-0.016	0.565
HN	0.004	0.447
AP	0.048	0.251
AK	-0.036	0.706

Table S3 Predicted top 20 metabolic pathways level 2 of bacterial community based on KEGG. The bold means the divergence is significant at 0.05 levels. Different uppercase letters indicate significant differences among different forest types.

Pathway level 1	Pathway level 2	Relative abundance (%)			*p* value
		P	B	N	
Metabolism	Amino acid metabolism	12.54 C	12.71 A	12.61 B	**0.000**
	Biosynthesis of other secondary metabolites	2.95 A	2.85 B	2.93 AB	**0.037**
	Carbohydrate metabolism	12.79	12.79	12.77	0.729
	Energy metabolism	5.26 A	5.22 B	5.26 A	**0.037**
	Glycan biosynthesis and metabolism	3.33 A	3.18 B	3.32 A	**0.027**
	Lipid metabolism	6.89 B	7.14 A	6.86 B	**0.018**
	Metabolism of cofactors and vitamins	11.88	11.90	11.92	0.907
	Metabolism of other amino acids	7.90	7.96	7.94	0.163
	Metabolism of terpenoids and polyketides	9.68	9.62	9.59	0.292
	Nucleotide metabolism	1.54	1.54	1.54	0.879
	Xenobiotics biodegradation and metabolism	6.02	6.33	6.12	0.235
Genetic Information Processing	Folding, sorting and degradation	3.29	3.24	3.26	0.291
	Replication and repair	4.96	4.95	5.00	0.242
	Transcription	0.84	0.84	0.84	0.987
	Translation	2.74	2.71	2.73	0.516
Environmental Information Processing	Membrane transport	1.55	1.58	1.57	0.168
	Signal transduction	0.43	0.42	0.43	0.109
Cellular Processes	Cell growth and death	1.45	1.44	1.45	0.276
	Cell motility	2.87 A	2.46 B	2.73 A	**0.000**
	Transport and catabolism	0.32 B	0.33 A	0.33 AB	**0.002**

P (Plantation forests); B (Bamboo forests); N (Natural forests).
