# Supplementary figures and images for: Impacts of Japanese Larch Invasion on Soil Bacterial Communities of the Giant Panda Habitat in the Qinling Mountains
Source: Microorganisms. 2022 Sep 9;10(9):1807. doi: 10.3390/microorganisms10091807 (PMC9500889; doi:10.3390/microorganisms10091807)

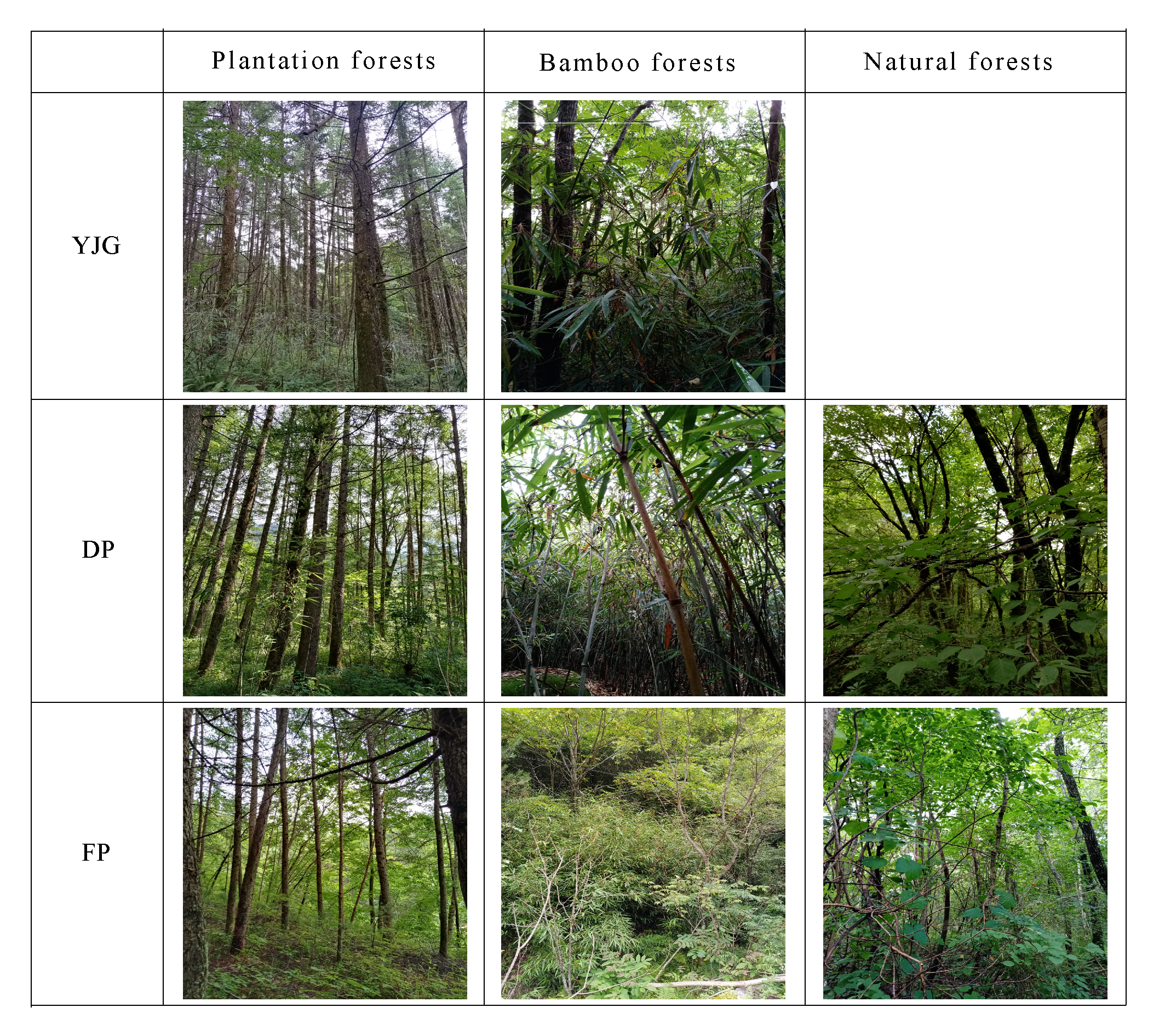

Supplement: Supplementary file 1 [file microorganisms-10-01807-s001.zip › Figure S1.tif]

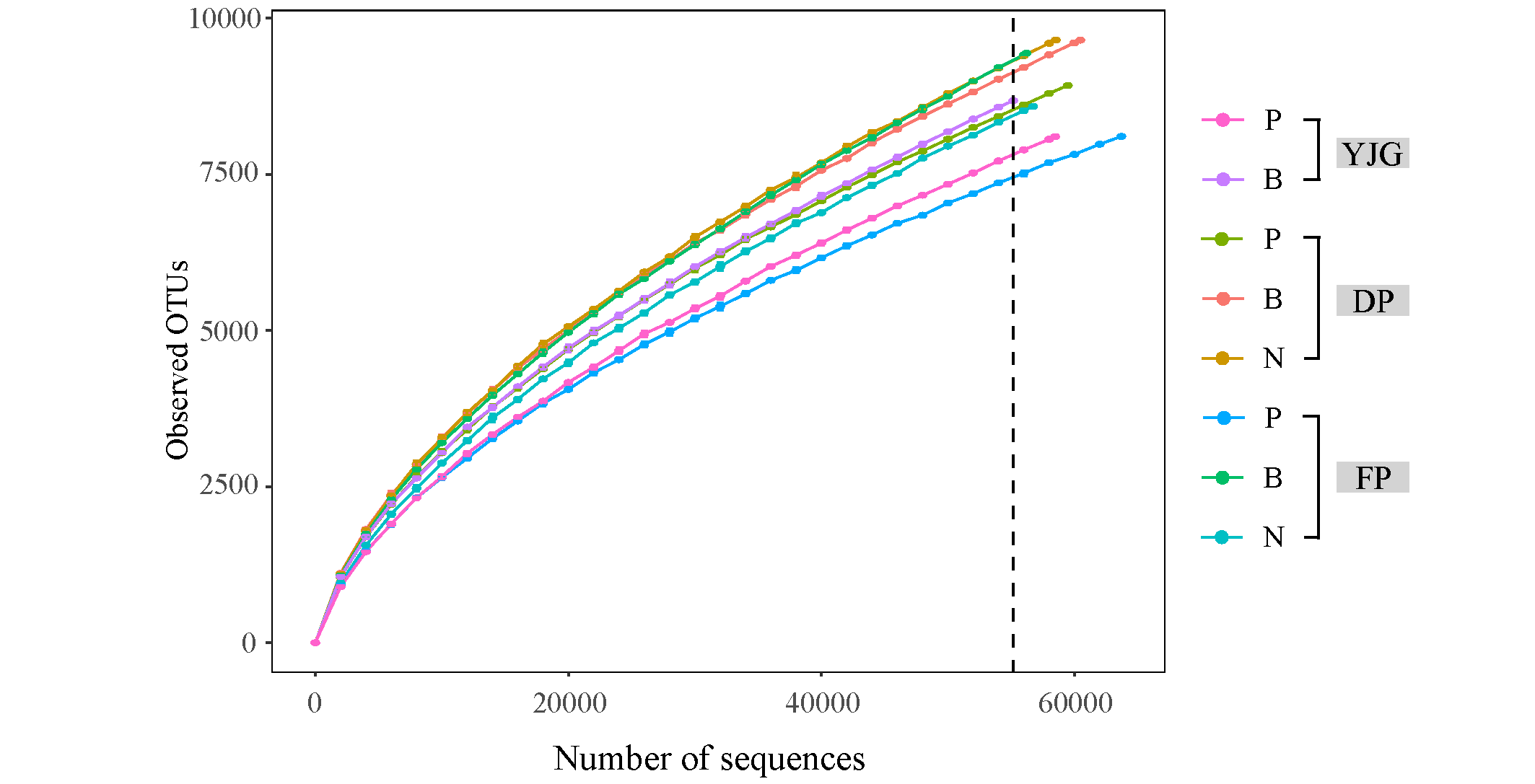

Supplement: Supplementary file 1 [file microorganisms-10-01807-s001.zip › Figure S2.tif]

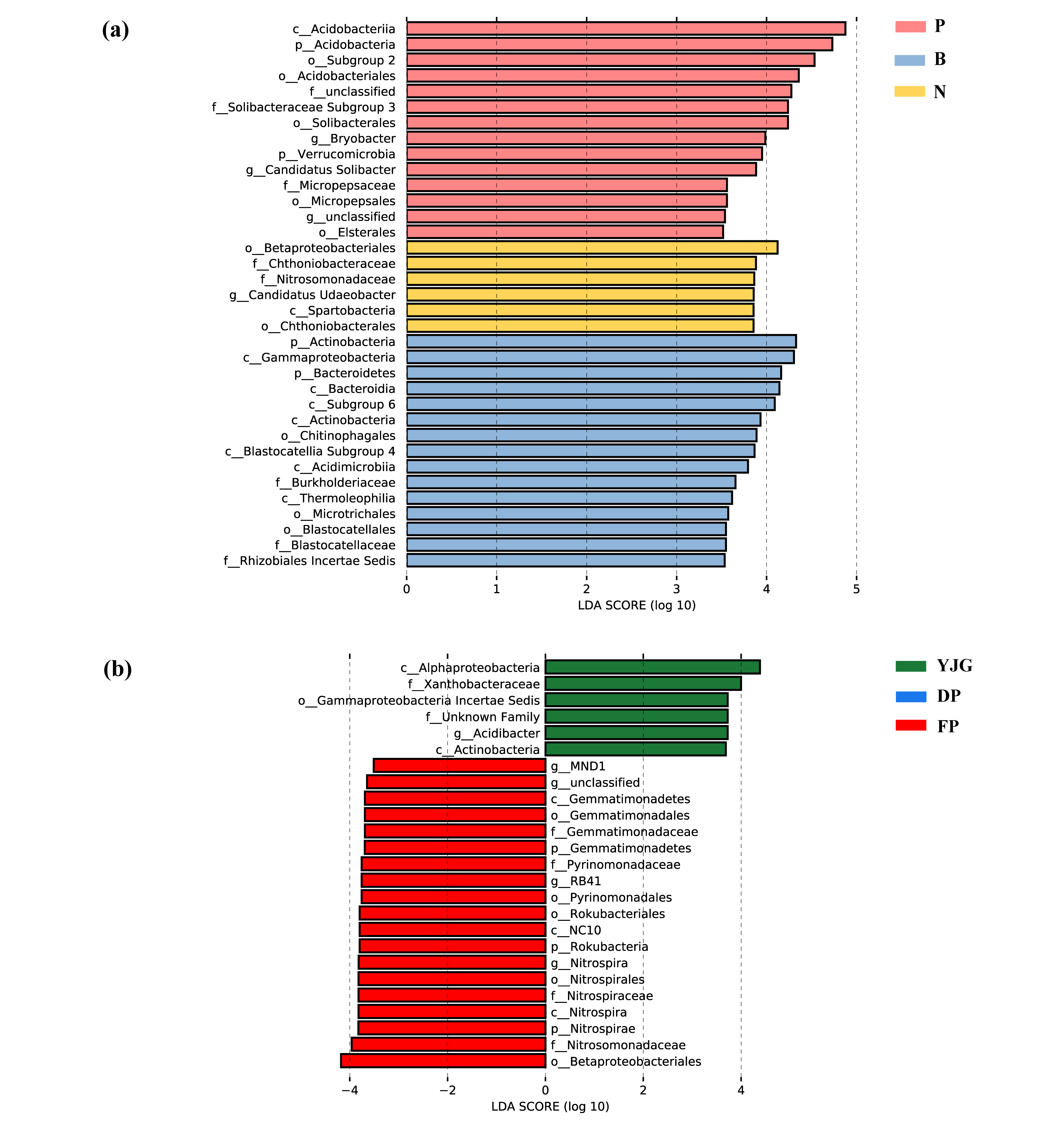

Supplement: Supplementary file 1 [file microorganisms-10-01807-s001.zip › Figure S3.tif]

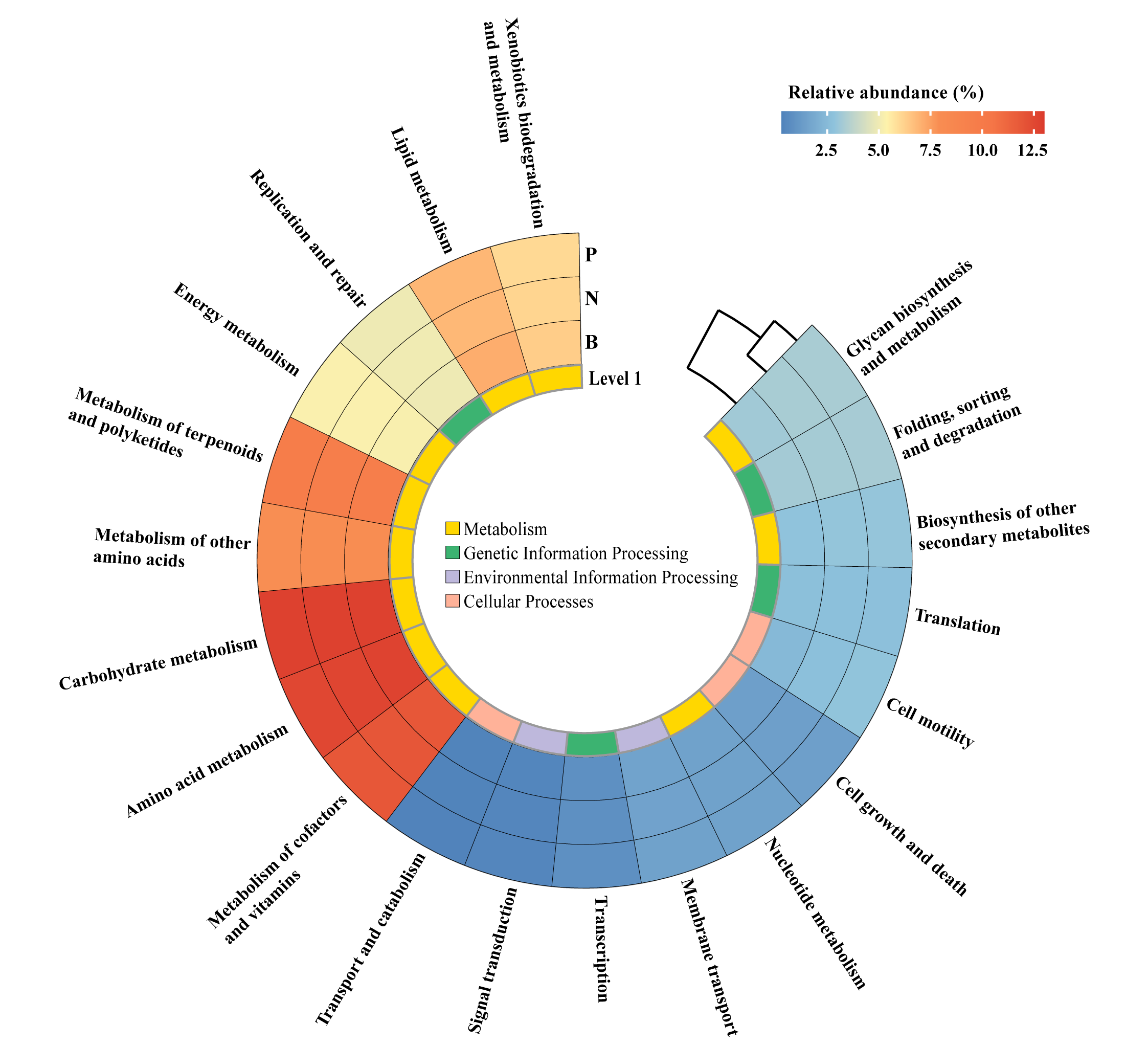

Supplement: Supplementary file 1 [file microorganisms-10-01807-s001.zip › Figure S4.tif]
